# A novel pyroptosis-related gene signature predicts the prognosis of glioma through immune infiltration

**DOI:** 10.1186/s12885-021-09046-2

**Published:** 2021-12-07

**Authors:** Moxuan Zhang, Yanhao Cheng, Zhengchun Xue, Qiang Sun, Jian Zhang

**Affiliations:** 1 Department of Neurosurgery, Linyi People’s Hospital, 27 Jiefang Road, Linyi, 276000 China; 2 Weifang Medical University, 7166 Baotong Road, Weifang, 261053 China

**Keywords:** Glioma, Pyroptosis, Prognostic, Immune, Tumor microenvironment

## Abstract

**Background:**

Glioma is the most common primary intracranial tumour and has a very poor prognosis. Pyroptosis, also known as inflammatory necrosis, is a type of programmed cell death that was discovered in recent years. The expression and role of pyroptosis-related genes in gliomas are still unclear.

**Methods:**

In this study, we analysed the RNA-seq and clinical information of glioma patients from The Cancer Genome Atlas (TCGA) database and Chinese Glioma Genome Atlas (CGGA) database. To investigate the prognosis and immune microenvironment of pyroptosis-related genes in gliomas, we constructed a risk model based on the TCGA cohort. The patients in the CGGA cohort were used as the validation cohort.

**Results:**

In this study, we identified 34 pyroptosis-related differentially expressed genes (DEGs) in glioma. By clustering these DEGs, all glioma cases can be divided into two clusters. Survival analysis showed that the overall survival time of Cluster 1 was significantly higher than that of Cluster 2. Using the TCGA cohort as the training set, a 10-gene risk model was constructed through univariate Cox regression analysis and LASSO Cox regression analysis. According to the risk score, gliomas were divided into high-risk and low-risk groups. Survival analysis showed that the low-risk group had a longer survival time than the high-risk group. The above results were verified in the CGGA validation cohort. To verify that the risk model was independent of other clinical features, the distribution and the Kaplan-Meier survival curves associated with risk scores were performed. Combined with the characteristics of the clinical cases, the risk score was found to be an independent factor predicting the overall survival of patients with glioma. The analysis of single sample Gene Set Enrichment Analysis (ssGSEA) showed that compared with the low-risk group, the high-risk group had immune cell and immune pathway activities that were significantly upregulated.

**Conclusion:**

We established 10 pyroptosis-related gene markers that can be used as independent clinical predictors and provide a potential mechanism for the treatment of glioma.

**Supplementary Information:**

The online version contains supplementary material available at 10.1186/s12885-021-09046-2.

## Introduction

Glioma accounts for most primary malignant brain tumours with high levels of mortality and aggressiveness in the central nervous system [1, 2]. As the most common type of central nervous system tumour, glioma has a poor treatment effect due to its easy recurrence and high mortality. Glioblastoma multiforme (GBM), or grade IV astrocytoma, is the most common malignant primary intracranial tumour and one of the most aggressive forms of brain cancer [3, 4]. Despite standard treatment comprising maximal surgical resection followed by radiotherapy and chemotherapy with alkylating agents such as temozolomide or some adjunct therapies, the clinical outcome remains dismal, with a median overall survival (OS) time of < 12 months for GBM [5, 6]. Considering the limitations of current glioma treatment, new treatment targets are needed to improve the clinical efficacy of glioma treatment and increase the survival time of patients. Therefore, scholars are continually exploring the mechanisms related to tumour occurrence, development and treatment to propose new clinical tumour prevention and treatment directions.

In recent years, great progress has been made in the molecular pathology of nervous system malignant tumours, and a series of molecular markers, such as MGMT promoter methylation, IDH mutations, chromosome 1p/19q co-deletion and TERT promoter mutations, have been found that are helpful for the clinical diagnosis and prediction of the formation, invasion, progression and prognosis of glioma [7, 8]. Among these molecular markers, IDH1, 1p19q, and MGMT methylation have been closely associated with the prognosis and chemotherapy sensitivity of glioma patients, although these markers have limited sensitivity and accuracy [9]. Therefore, it is urgent that reliable new prognostic models be developed to make targeted therapy more feasible.

Pyroptosis, also known as cell inflammatory necrosis, is a new type of programmed cell death that manifests as cell swelling until the cell membrane ruptures, which leads to the release of cell contents and activates a strong inflammatory response [10, 11]. Pyroptosis plays a key role in anti-infection and immune defence [12–14]. Pyroptosis was discovered in 1992 by Zychlinsky et al. in macrophages infected by Shigella [15]. Shigella-induced host cell death was originally described as apoptosis [15]. Subsequent studies revealed that this kind of apoptosis is a pathway of cell death that does not depend on the apoptotic executive factor caspase-3 but on the activity of inflammatory caspase-1 [16]. In 2001, Cookson et al. named this type of cell death dependent on inflammasomes pyroptosis [17]. Pyroptosis is characterized by rupture of the cell membrane, the formation of inflammasomes, and the release of inflammatory factors, which ultimately leads to cell death [18]. When cells are stimulated, intracellular inflammatory bodies are formed, which in turn activates Caspase to cleave the Gasdermin (GSDM) protein family, release the N-terminal domain to recognize the cell membrane and form a 10 ~ 15 nm Tunnel [19]. This pore-forming activity destroys the osmotic pressure of the cell, and the imbalance of the electrolyte inside and outside the cell membrane causes the cell to swell and rupture, release a large amount of inflammatory factors and cell contents, recruit immune cells to further expand the inflammatory response, and ultimately lead to inflammatory cell death [12, 13]. The GSDM protein family is a group of important proteins that mediate pyroptosis and play an important role in inducing cell death and inflammation. The GSDM protein family consists of gasdermin A (GSDMA), gasdermin B (GSDMB), gasdermin C (GSDMC), gasdermin D (GSDMD), gasdermin E (GSDME) and DFNB59 [12]. The molecular mechanism of GSDM family members inducing cell pyrolysis is considered to be the N-terminal domain using different molecular interaction mechanisms to form pores in the cell membrane [20–23]. Due to the importance of the GSDM family in the process of cell pyrolysis, the Nomenclature Committee on Cell Death (NCCD) in 2018 defined cell pyrolysis as programmed cell death mediated by the Gasdermin protein family [24].

Early studies of pyroptosis were based on infection models, including infectious diseases such as Shigella freundii, *Streptococcus pneumoniae*, drug-resistant *Staphylococcus aureus*, and human immunodeficiency virus (HIV) [25–27]. In recent years, studies of neurological diseases have also found that cerebral ischaemia, brain injury, Parkinson’s disease, Alzheimer’s disease and other diseases are all related to the classic pyroptosis mediated by Caspase-1 [28]. With the deepening of research, the role of pyroptosis in tumours has become increasingly prominent. Scholars have discovered that cell pyroptosis may play a double-edged role in the occurrence and treatment of tumours. On the one hand, when normal cells are stimulated to undergo pyroptosis, they release a large number of inflammatory factors, forming an inflammatory microenvironment and causing normal cells to transform into tumour cells [29]. On the other hand, the induction of pyroptosis in tumour cells can be used as a new therapeutic target to inhibit the occurrence and development of tumours [30].

Although the role of pyroptosis in tumours has received increasing attention, the specific mechanism of pyroptosis and its role in tumours deserve further study. In this study, we evaluated the expression level of pyroptosis-related genes in gliomas and developed a pyroptosis-related risk signature module to predict glioma patient prognosis. Importantly, we verified the signature of pyroptosis-related genes, which can improve accurate prediction of the prognosis of gliomas.

## Materials and methods

### Datasets

**Fig. 1:**
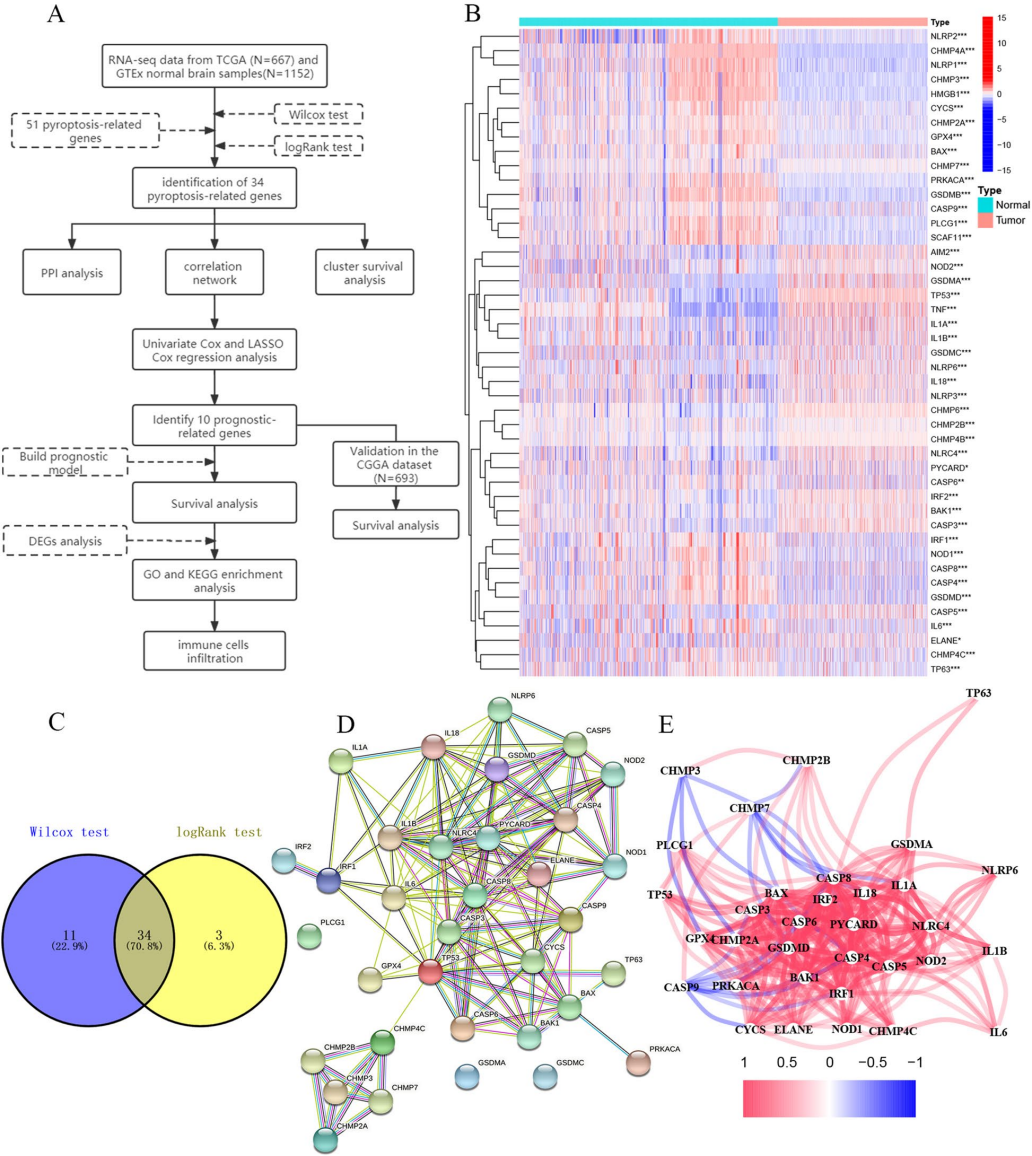
Experimental flow chart and identification of pyroptosis-related genes in gliomas. **A** Experimental flow chart. **B** DEGs heatmap of normal tissue and tumour tissue. (*P < 0.05; **P < 0.01; * * * *P* < 0.001) **C** Venny graph of the Wilcox test and log-rank test. **D** The PPI network showed the interaction of DEGs. **E** The correlation network of DEGs (red line: positive correlation; blue line: negative correlation. The depth of the colour represents the strength of the correlation)

We obtained RNA sequencing (FPKM) data and corresponding clinical characteristics of 667 glioma patients from the TCGA database on 29 July 2021, including 509 LGG and 158 GBM patients (https://portal.gdc.cancer.gov/). Patients without survival information were excluded from further analysis. The RNA sequencing data (FPKM) of 1152 normal brain samples were collected from the GTEx database downloaded from Xena on 29 July 2021 (http://xena.ucsc.edu/). We downloaded the RNA sequencing data and corresponding clinical characteristics of 693 glioma patients from the CCGA database (http://www.cgga.org.cn/index.jsp). The research flow chart is shown in Fig. 1A.

### Identification of differentially expressed pyroptosis-related genes

We obtained 29 pyroptosis genes from the GSEA website (https://www.gsea-msigdb.org/gsea/). Then, we discovered 33 pyroptosis genes from previous studies [29–33]. By taking the union, we obtained 51 pyroptosis-related genes, which are shown in Supplementary Table 1. The “combat” function in the “sva” package was used on TCGA and GTEx data to normalize RNA expression profiles and to remove batch effects. Combat algorithms used parametric for adjusting data for batch effects. By dividing 667 gliomas and 1152 normal brain tissues into two groups, DEGs identification was performed using Wilcox test in the “limma” package with a *P* < 0.05. Then, the DEGs were retested by the log-rank (*P* < 0.05) test to determine the final DEGs.

### Construction and analysis of PPI network

The STRING website (http://string-db.org/cgi/) is a visual tool that evaluates protein-protein interaction information. To analyze the relationship between the pyroptosis-related genes, the protein-protein interaction (PPI) network was built using the STRING online tool and the interaction score > 0.4 was chosen as the cutoff criterion.

### Consensus clustering and gene set variation analysis (GSVA)

In order to show whether pyroptosis has an important impact on the overall prognosis of patients with gliomas, consensus clustering was performed to divide patients into clusters based on the pyroptosis-related genes. “ConsensusClusterPlus” package in R was employed for consensus clustering for identifying the subgroups based on the pyroptosis-related genes [34]. The values of k were chosen as the optimal number of clusters based on where the magnitude of the cophenetic correlation coefficient began to fall. Gliomas were sampled using paritioning around medoids algorithm as well as squared Euclidean distance metric. About 80% of the samples were selected in each iteration, and the results were compiled over 50 iterations. “GSVA” package was used to perform GSVA between Cluster 1 and Cluster 2, using the hallmark gene sets as a reference [35]. We set FDR < 0.05 as the cut-off values to identify signal pathways.

### Prognostic signature

The prognostic value of pyroptosis-related genes in the TCGA training cohort was determined by univariate Cox regression analysis. The hazard ratio (HR) from univariate Cox regression analysis was used to identify candidate genes related to overall survival from the TCGA cohort. HR < 1 is a protective gene, and HR > 1 is a risk gene. *P* < 0.05 was considered statistically significant. Then, the Least absolute shrinkage and selection operator (LASSO) Cox regression model (“glmnet” package) was used to narrow down the overfitting candidate genes and build a prognostic model. Finally, a multivariate Cox regression analysis was performed to identify highly correlated genes and construct the prognostic signature. The regression coefficient (β) was derived from multivariate Cox regression analysis. The expression value of the candidate gene was combined with their regression coefficient to weight, and the risk score of each patient was constructed as follows:

$$Risk\kern0.5em score=\sum \limits_{i=1}^n{\exp}_i^{\ast}\kern0.5em \beta i$$

where n is the number of prognostic genes, exp_i_ is the expression value of gene i, and βi is the regression coefficient of gene i in multivariate Cox regression analysis. With the median risk score as the critical value, glioma patients were divided into high-risk and low-risk groups. The validation group was used to externally verify the prognostic ability of the risk characteristics of pyroptosis genes. The validation group data and clinicopathological information were from the CGGA database [36]. The validation group removed the batch effect through the “combat” function in the “sva” package of R. Using the median risk score of the TCGA cohort, the patients in the validation group were also divided into high-risk and low-risk. Next, Kaplan-Meier survival curves and log-rank tests were used to evaluate the survival rate of the low-risk and high-risk groups in the training group and the validation group. The time-dependent receiver operating characteristic curve (ROC) curve was drawn based on the risk score from the “survival”, “survminer” and “timeROC” R packages. The area under the curve (AUC) represents the 1-year, 3-year, and 5-year OS probability to estimate the accuracy of the actual observation rate and the predicted survival probability. Principal component analysis (PCA) based on the 10-gene signature was performed with the “ggplot” package through the “prcomp” function.

### Independent prognostic analysis of risk scores based on the TCGA and CGGA datasets

We extracted and merged the clinical information of patients in the TCGA and CGGA databases. These variables were analysed in combination with the risk score in the regression model. Univariate and multivariate Cox regression models were used for exploratory independent prognostic analysis. The “forestplot” package in R was used to obtain multivariate prognostic analysis results, including risk scores. Forest plots were used to show the results of univariate and multivariate Cox regression analyses. A nomogram constructed with several independent indicators can be used to predict patient prognosis. To assess the accuracy of the nomogram, a calibration curve was used to predict 1-year, 3-year, and 5-year OS.

### Identification of DEGs in the low-risk and high-risk groups from the TCGA dataset

The Wilcox test in the “limma” package was used to screen significantly differentially expressed genes between the high-risk and low-risk groups in the TCGA cohort. The cut-off values for DEG screening were based on |log_2_FC| ≥ 2 and FDR < 0.05.

### Functional enrichment analysis of DEGs based on low-risk and high-risk

Gene Ontology (GO) and Kyoto Encyclopedia of Genes and Genomes (KEGG) analyses based on DEGs were performed with the “clusterProfiler” package. Next, we used the “gsva” package to perform ssGSEA, calculate the score of infiltrating immune cells, and evaluate the immune-related pathways.

## Results

### Identification of differentially expressed pyroptosis genes in gliomas and normal brain tissues

The 51 pyroptosis-related genes expression were compared in 1152 normal brain tissues (GTEx) and 667 glioma tissues (TCGA), and we identified 45 differentially expressed genes (DEGs) (all *P* < 0.05) (Fig. 1B). Then through a log-rank test, a total of 37 prognostic difference genes were identified from 51 pyroptosis-related genes (all *P* < 0.05). Taking their intersection, a total of 34 hub genes for further analysis (Fig. 1C). Among them, 15 genes (IL1A, GSDMA, TP53, NLRP6, GSDMC, IL1B, NLRC4, IL18, CASP5, CASP3, IRF2, BAK1, PYCARD, CHMP2B, and NOD2) were upregulated, while 19 other genes (CASP6, CHMP7, ELANE, BAX, CHMP2A, CASP9, GPX4, CYCS, CASP8, PRKACA, TP63, IRF1, PLCG1, CHMP3, GSDMD, CHMP4C, NOD1, CASP4, and IL6) were downregulated in the glioma group. To further explore the interaction between these genes related to pyroptosis, we conducted PPI analysis, and the results showed that IL1B, CASP8, TP53, PYCARD, IL18 and IL6 were the core genes (degree score > 15) (Fig. 1D). As shown in Fig. 1E, the relevant network of all differential pyroptosis genes in TCGA (red: positive correlation; blue: negative correlation).

### Clustering of tumour samples based on hub genes

**Fig. 2:**
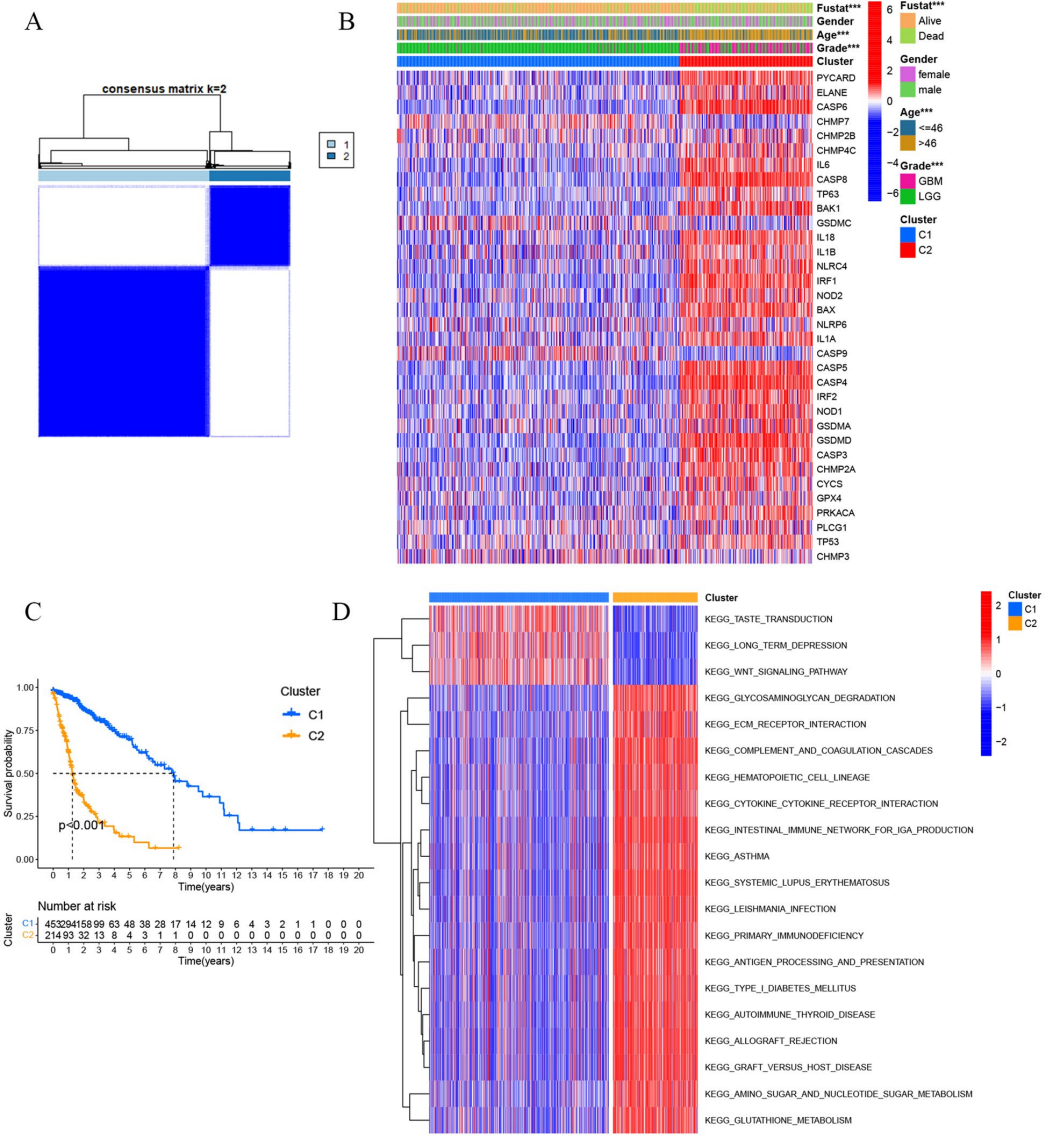
Cluster of differentially expressed pyroptosis-related genes. TCGA patients were divided into two clusters according to the consensus score matrix (k = 2). **B** The two cluster heatmaps based on DEGs with clinical characteristics, including sex, age and pathological grade (LGG: low-grade glioma; GBM: glioblastoma multiforme). **C** Survival curves of the two clusters. **D** Heatmapping was used to visualize the biological process by GSVA analysis in the 2 clusters

To explore the relationship between the hub genes and overall survival, we performed a cluster analysis of all 667 glioma patients in the TCGA cohort. By increasing the clustering variable (k) from 2 to 9, we found that when k = 2, the correlation within the group was highest, and 667 glioma patients could be well divided into two cluster (Fig. 2A). Gene expression and clinical characteristics, including sex, age, grade and survival status, were all shown in one heat map, and we found significant differences in age, grade and survival status between the two cluster (*P* < 0.05) (Fig. 2B). The overall survival time (OS) of Cluster 1 was significantly higher than that of Cluster 2 (*P* < 0.01) (Fig. 2C). To explore the biological differences between these two clusters, we performed a GSVA enrichment analysis (Fig. 2D). Cluster 1 showed enrichment of carcinogenic activation pathways, such as the WNT signalling pathway. Cluster 2 showed the enrichment of immune-related pathways, including ECM receptor interaction, complement and coagulation cascades, antigen processing and some immune-related diseases (Supplementary Table 2).

### Establishment of a prognostic model in the TCGA cohort

**Fig. 3:**
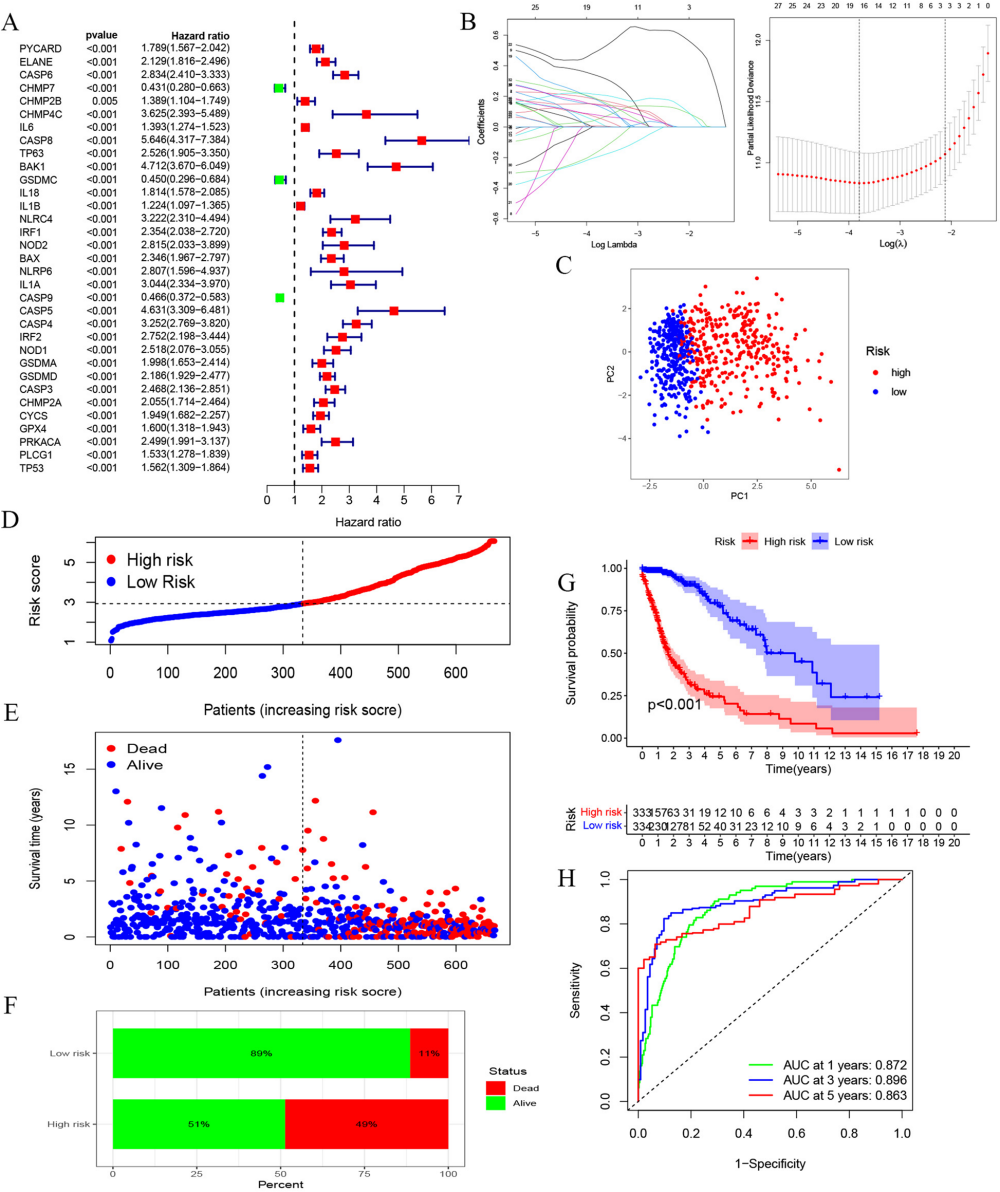
Establishment of a risk model based on the TCGA cohort. **A** Univariate cox regression analysis of DEGs. **B** LASSO regression of the DEGs and the tuning parameter (λ) selection cross-validation curve. **C** PCA plot for TCGA patients based on the risk score. **D** Distribution of risk scores for glioma patients in the TCGA cohort. **E** The distribution of patient status according to low-risk and high-risk groups (the dots represent the status of the patient, sorted by the increase in risk score. Low-risk cases: the left side of the dotted line; high-risk cases: the right side of the dotted line). **F** Mortality rates among the high-risk and low-risk groups. **G** Kaplan-Meier curve of the high-risk and low-risk groups. **H** The ROC curve shows the predictive efficiency of the risk model

A total of 667 glioma samples were matched with corresponding survival information. Univariate Cox regression analysis was used to preliminarily screen 34 pyroptosis-related genes. The results showed that 33 genes were statistically significant for further analysis (*P* < 0.01) (Fig. 3A). Among them, 30 genes with HR > 1 were associated with increased risk, while the other 3 genes with HR < 1 were protective genes (CHMP7, CSDMC, CASP9). To establish a model that can quantify each patient, the LASSO-Cox regression model was used to retain 17 of the 33 DEGs, with a minimum value of λ (Fig. 3B). λ is called the regularization parameter, which gives the most regularized model so that the cross-validation error is within one standard error of the minimum value. These genes entered the multivariable Cox analysis, and 10 genes and correlation coefficients were obtained. The risk score was calculated by: Risk Score = (0.233*ELANE exp.) + (0.406*TP63 exp.) + (− 0.385*GSDMC exp.) + (0.261*IL18 exp.) + (0.419*IL1A exp.) + (− 0.322*CASP9 exp.) + (0.493*CASP4 exp.) + (0.264*CASP3 exp.) + (0.178*CYCS exp.) + (0.351*PLCG1 exp.). Principal component analysis (PCA) showed that high-risk and low-risk patients could be divided into two groups (Fig. 3C). According to the median score calculated by the risk score, 667 glioma patients were equally divided into low-risk and high-risk subgroups (Fig. 3D). Figure 3E and F shows that the number of deaths increased in the high-risk group compared with the low-risk group. The high-risk group had a shorter overall survival time than the lower-risk group, and the difference was statistically significant (*P* < 0.01) (Fig. 3G). Using ROC analysis to evaluate the specificity and sensitivity of the prognostic model, we found that the area under the ROC curve (AUC) was 0.872 at 1 year, 0.896 at 2 years, and 0.863 at 3 years (Fig. 3H).

### Validation of the prognostic risk model

**Fig. 4:**
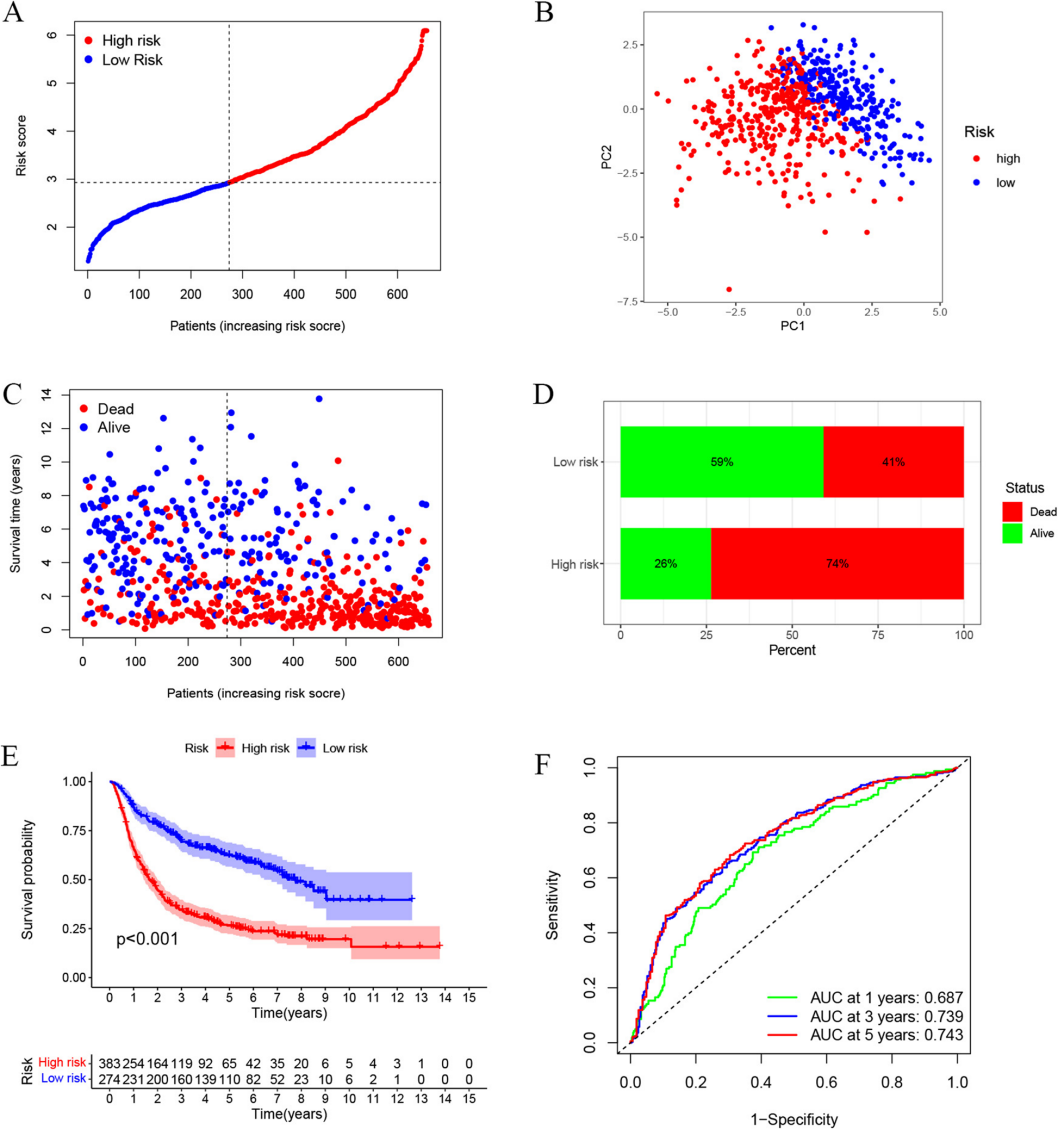
Verification of the risk model in the CGGA cohort. **A** Distribution of risk scores for glioma patients in the CGGA cohort. **B** PCA plot for CGGA patients based on the risk score. **C** The distribution of patient status in the CGGA cohort (the dots represent the status of the patient, sorted by the increase in risk score). Low-risk cases: the left side of the dotted line; high-risk cases: the right side of the dotted line). **D** Mortality rates among the high-risk and low-risk groups. **E** Kaplan-Meier curve of the high-risk and low-risk groups. **F** The ROC curve shows the predictive efficiency of the risk model

The 693 glioma patients in the CGGA dataset were used for independent verification to further test the predictive value of the risk score. According to the median risk score of the TCGA cohort, 274 patients in the CGGA cohort were allocated to the low-risk group, and 383 patients were allocated to the high-risk group (Fig. 4A). PCA showed that high-risk and low-risk patients were well divided into two groups (Fig. 4B). Patients in the low-risk group had a longer survival time and a lower mortality rate than those in the high-risk group (Fig. 4C and D). Kaplan-Meier analysis also showed significant differences in survival time between the low-risk and high-risk groups (*P* < 0.01) (Fig. 4E). The ROC analysis showed that the AUCs of the signature for the prediction of 1-, 3-, and 5-year OS were 0.687, 0.739 and 0.743, respectively (Fig. 4F).

### Stratifification analysis of the risk models

**Fig. 5:**
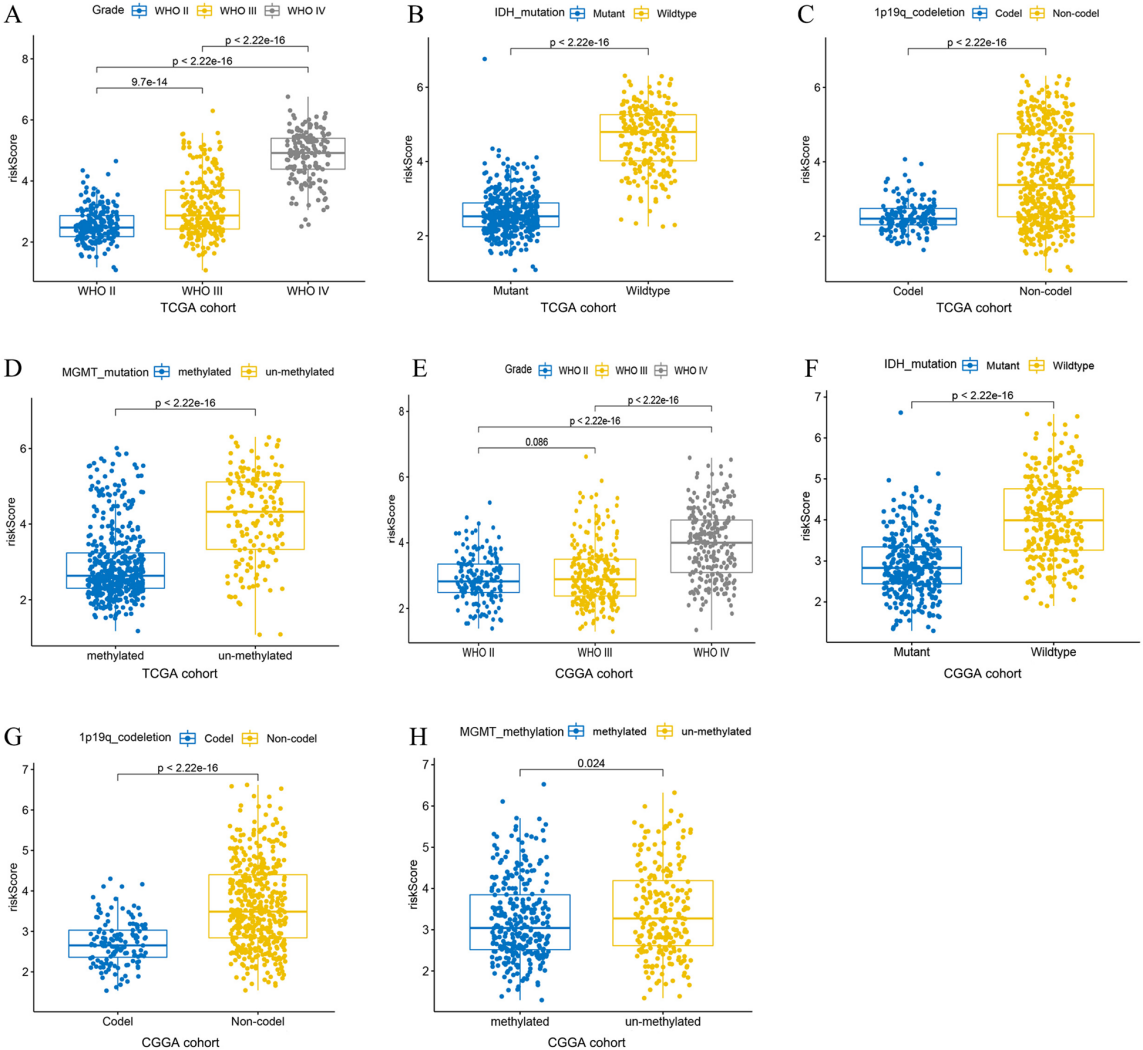
Correlation between risk score and different subtypes. **A** Risk score distribution between different WHO grade in TCGA cohort. **B-D** Distribution of risk scores among different molecular subtypes in TCGA cohort. **E** Risk score distribution between different WHO grade in CGGA cohort. **F-H** Distribution of risk scores among different molecular subtypes in CGGA cohort

**Fig. 6:**
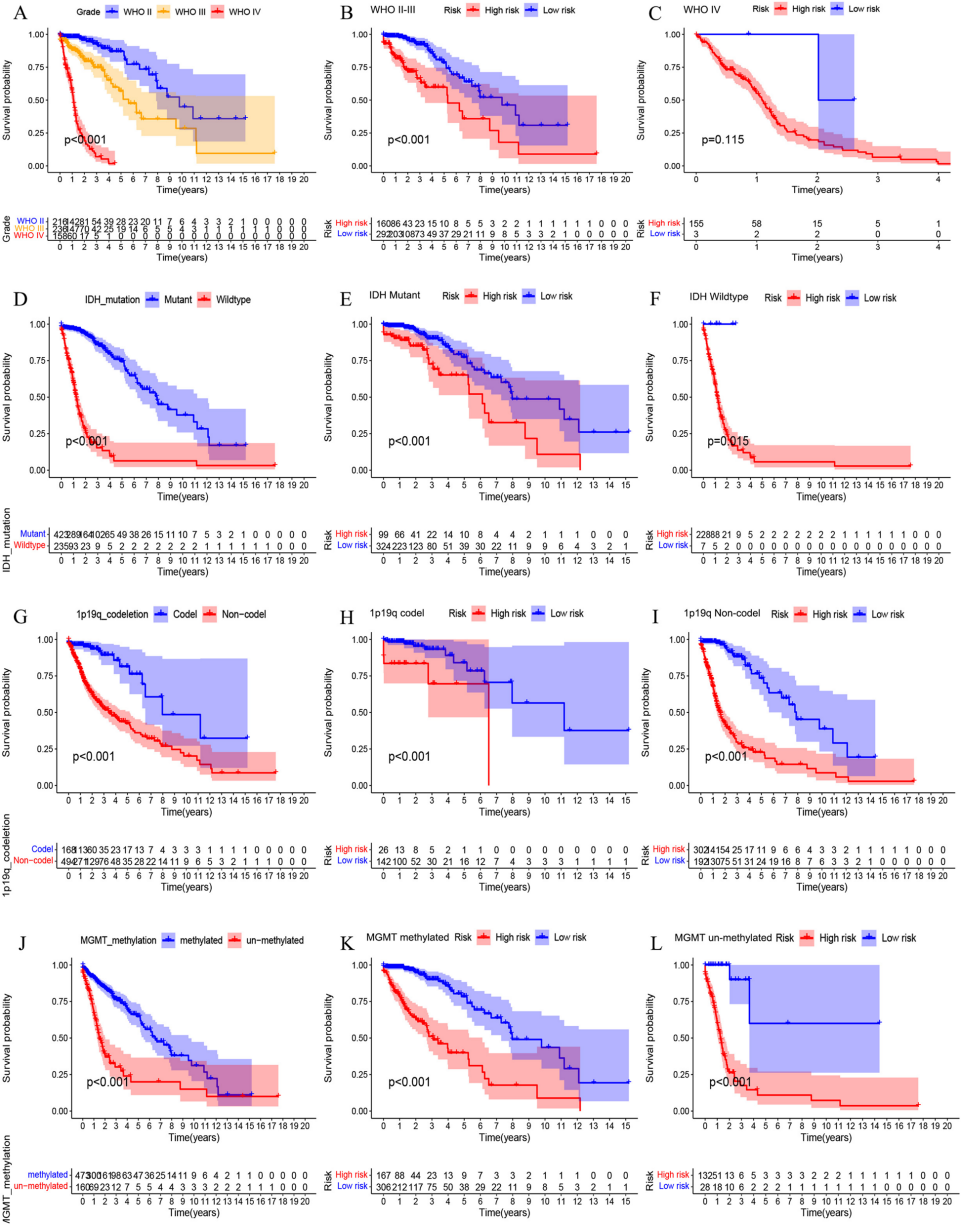
Kaplan-Meier survival subgroup analysis according to the signature stratified by different subtypes in TCGA cohort. **A** Grade. **B** WHO II-III. **C** WHO IV. **D** IDH mutation. **E** IDH Mutant. **F** IDH Wildtype. **G** 1p19q codeletion. **H** 1p19q Codel. **I** 1p19q Non-codel. **J** MGMT methylation. **K** MGMT methylated. **L** MGMT un-methylated

**Fig. 7:**
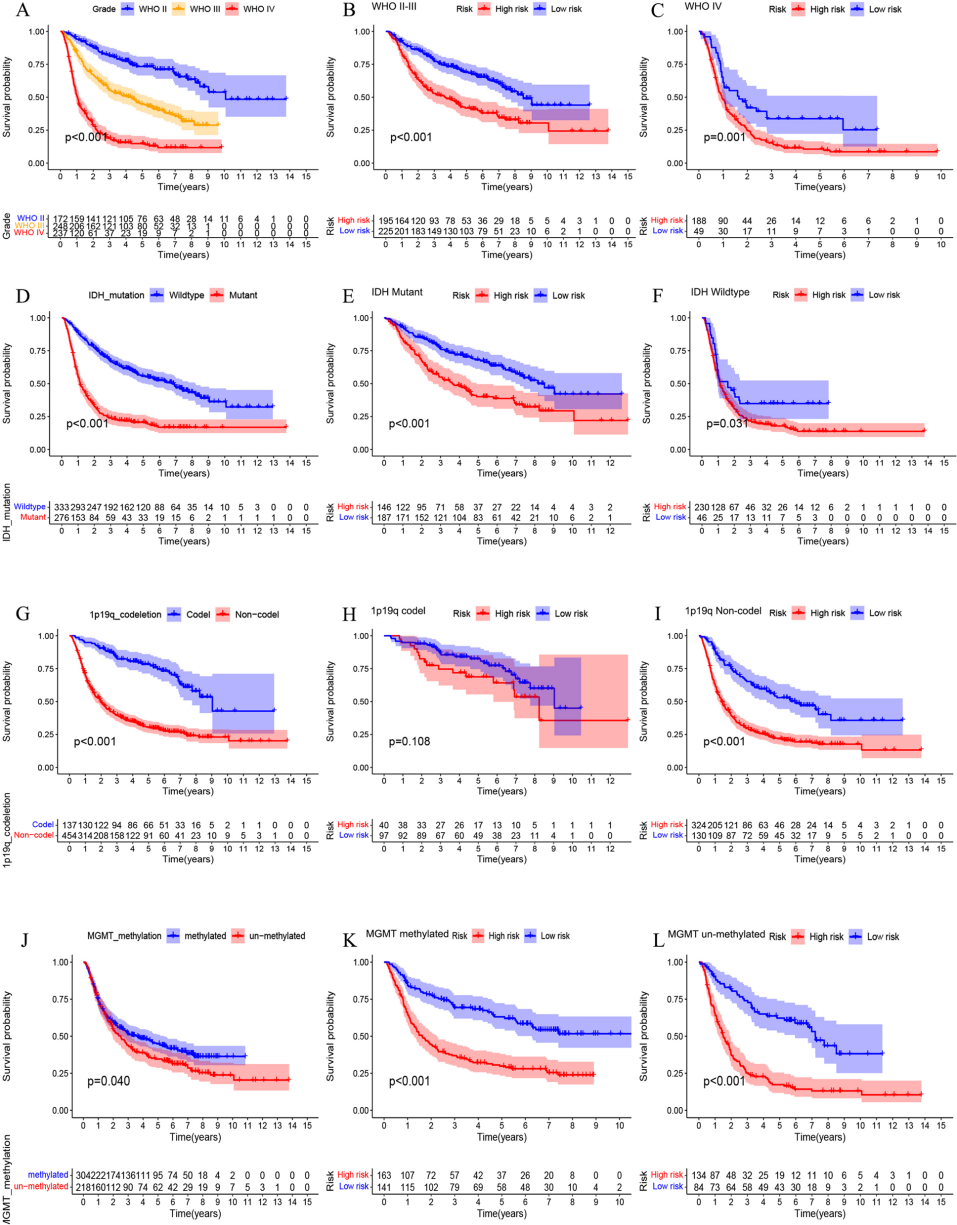
Kaplan-Meier survival subgroup analysis according to the signature stratified by different subtypes in CGGA cohort. **A** Grade. **B** WHO II-III. **C** WHO IV. **D** IDH mutation. **E** IDH Mutant. **F** IDH Wildtype. **G** 1p19q codeletion. **H** 1p19q Codel. **I** 1p19q Non-codel. **J** MGMT methylation. **K** MGMT methylated. **L** MGMT un-methylated

In order to conduct a stratified analysis, we evaluated the prognostic value of the risk model in the TCGA cohort and the CGGA cohort in different groups. As shown in Fig. 5A, we found that the risk score is positively correlated with the pathological grade in the TCGA cohort. The TCGA cohort was divided into two groups by different molecular models including IDH mutation, 1p19q co-deletion and MGMT methylation. The results showed that the risk score of IDH wild-type patients was significantly higher than that of mutant patients (Fig. 5B). Patients with 1p19q non-codel have significantly higher risk scores than 1p19q codel patients (Fig. 5C). The risk score of MGMT un-methylated patients was significantly higher than that of MGMT methylated patients (Fig. 5D). The distribution of risk scores among different molecular types was verified in the CGGA cohort (Fig. 5 E-H). Subsequently, we performed survival analysis to assess the survival differences in risk scores between different subgroups. The results showed that in the TCGA cohort, the overall survival of patients with high-risk scores was shorter than that of patients with low-risk scores (Fig. 6). In particular, there is no statistical difference in WHO IV, which is considered to be due to the small number of samples in the low-risk group. The above results were verified in the CGGA cohort (Fig. 7).

### Evaluate the independent prognostic value of risk models

**Fig. 8:**
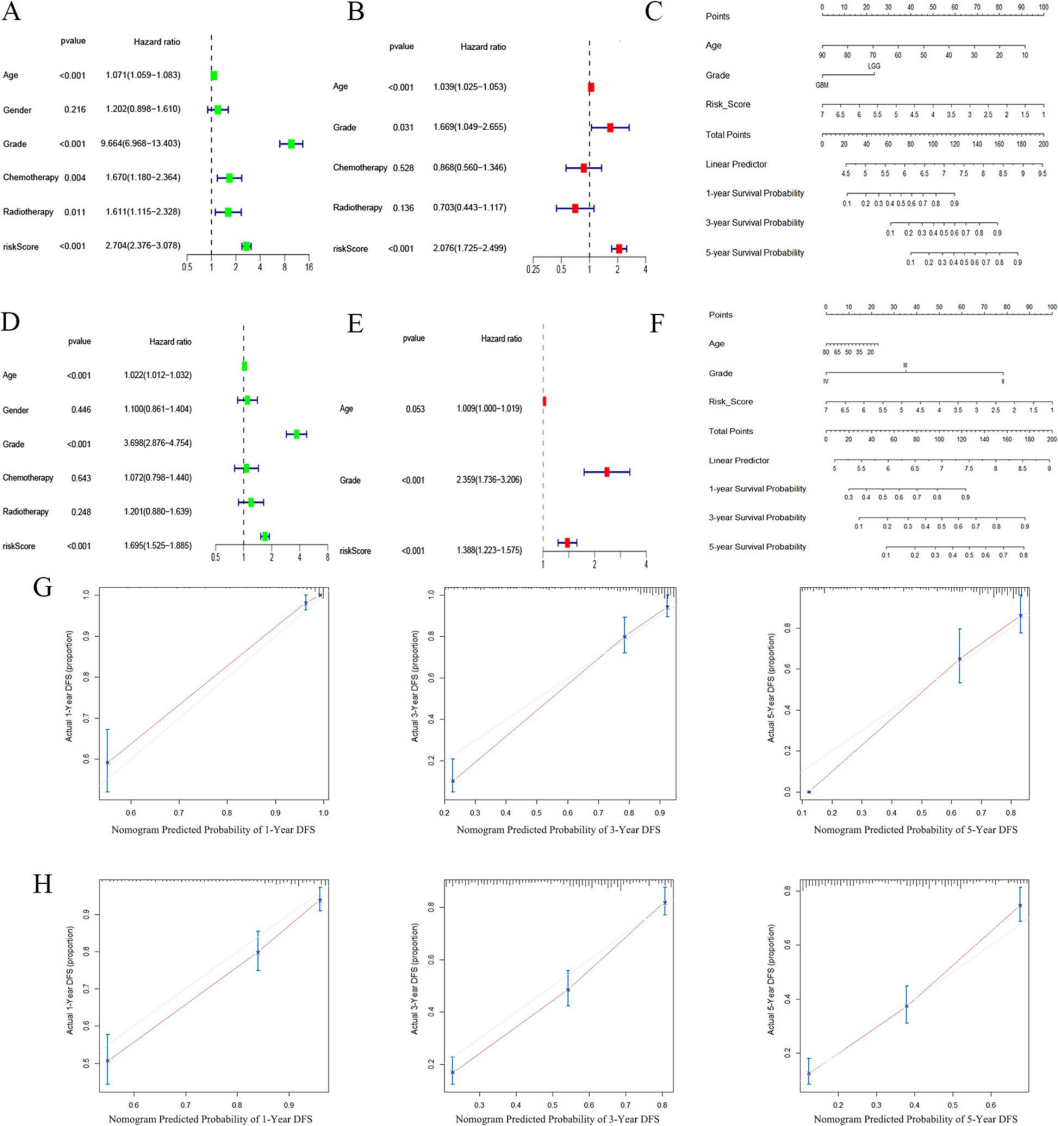
Univariate and multivariate Cox analyses of the risk score and clinical characteristics. **A** Univariate cox analysis of the TCGA cohort. **B** Multivariate Cox analysis of the TCGA cohort. **C** The nomogram for predicting the proportion of patients with 1-, 3- and 5-year overall survival in the TCGA cohort. **D** Univariate Cox analysis of the CGGA cohort. **E** Multivariate Cox analysis of the CGGA cohort. **F** The nomogram for predicting the proportion of patients with 1-, 3- and 5-year overall survival in the CGGA cohort. **G** Calibration curve for the prediction of 1-, 3- and 5-year overall survival in the TCGA cohort. **H** Calibration curve for the prediction of 1-, 3- and 5-year overall survival in the CGGA cohort

**Fig. 9:**
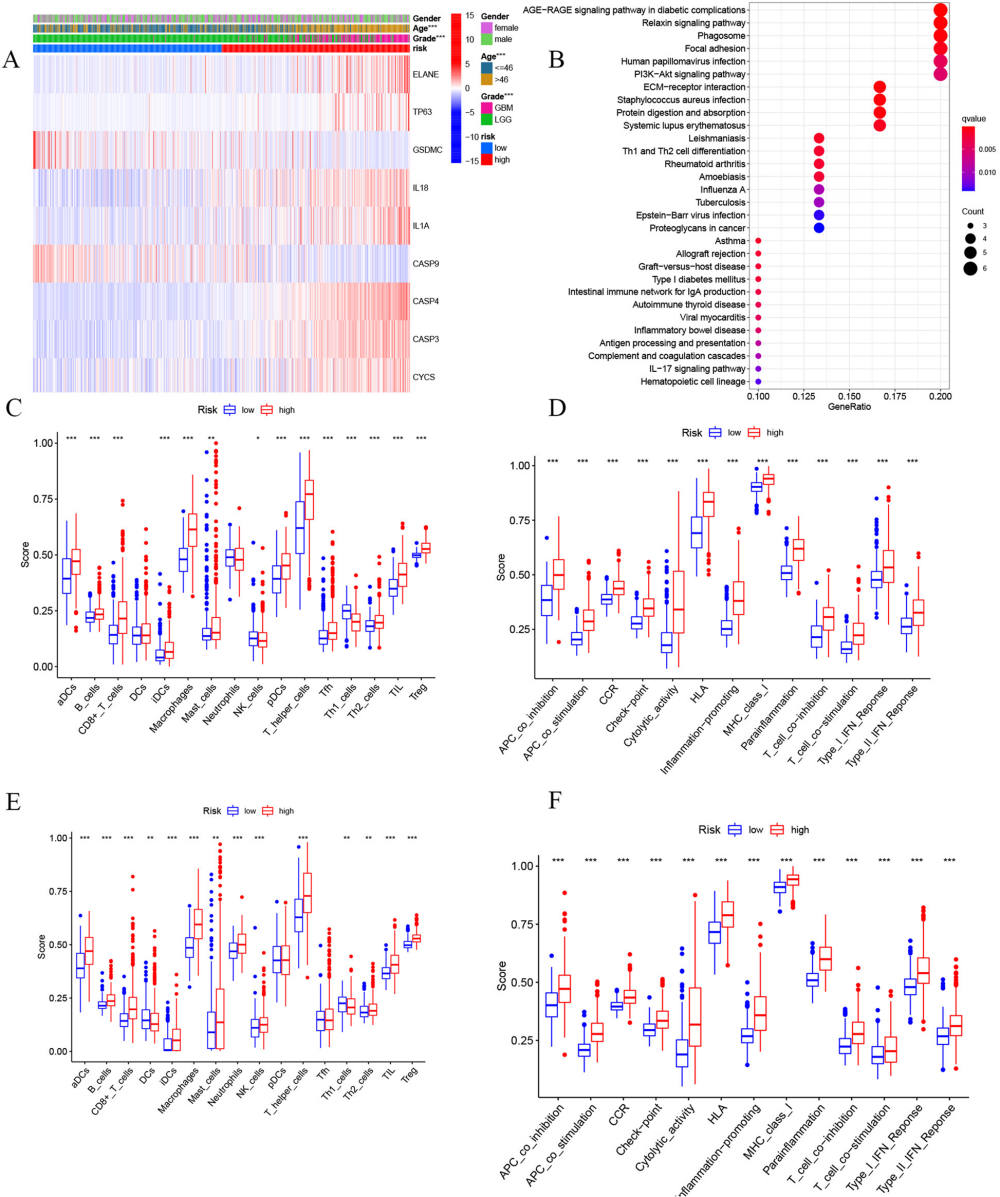
Functional analysis and immune analysis for the two risk groups. **A** 10-signature gene heatmap in the TCGA cohort (* * * *P* < 0.001). **B** Bubble graph for KEGG enrichment analysis. **C-D** The enrichment scores of 16 immune cells and 13 immune-related pathways for the low-risk and high-risk groups in the TCGA cohort. **E-F** The enrichment scores of 16 immune cells and 13 immune-related pathways for the low-risk and high-risk groups in the CGGA cohort

We incorporated the risk score and other clinical characteristics into the Cox regression analysis. Univariate and multivariate Cox analyses were used to determine the independent prognostic value of the risk score. Univariate Cox regression analysis showed that in TCGA and CGGA, the risk score was a factor to predict poor survival (TCGA: HR = 2.704, 95% CI: 2.376–3.078; CGGA: HR = 1.695, 95% CI: 1.525–1.885) (Fig. 8A and D). Multivariate Cox regression analysis showed that the risk score was an independent prognostic factor in TCGA and CGGA (TCGA: HR = 2.076, 95% CI: 1.725–2.499; CGGA: HR = 1.388, 95% CI: 1.223–1.575) (Fig. 8B and E). Among them, in the TCGA cohort, age and WHO grade were also independent prognostic factors (*P* < 0.05). In the CGGA cohort, WHO grade was also an independent prognostic factor (*P* < 0.05). We then selected three independent prognostic factors and used them to construct a nomogram that predicted overall survival probabilities at 1, 3, and 5 years (Fig. 8C and F). Each prognostic parameter had a score, and the sum of the three prognostic parameter scores was used to predict the probability of 1-year, 3-year, and 5-year overall survival. In addition, to determine the prediction effect of the nomogram, we used the calibration curve to judge the effect of the nomogram, and the results showed that the nomogram prediction accuracy was better (Fig. 8G-H). In addition, we drew a heatmap of the clinical characteristics of the TCGA cohort, and the results showed that the patient’s age and pathological grade were different in the distribution between the low- and high-risk subgroups (*P* < 0.05) (Fig. 9A).

### GO and KEGG enrichment analysis of risk model

To further explore the differences in the biological functions and pathways involved between risk models, we identified DEGs between the high-risk group and the low-risk group. A total of 58 DEGs between the low-risk group and high-risk groups were determined. Among them, 49 genes were upregulated in the high-risk group, and 9 genes were downregulated. Then, based on these DEGs, Kyoto Encyclopedia of Genes and Genomes (KEGG) enrichment analysis was performed. The results showed that DEGs were significantly enriched in immune-related pathways such as the phagosome pathway, Th1 and Th2 cell differentiation and some immune-related diseases (Fig. 9B) (Supplementary Table 3).

### The relationship between risk score and the immune microenvironment

On the basis of functional analysis, we used single-sample gene set enrichment analysis (ssGSEA) to further compare the enrichment scores of immune cells and the activities of immune-related pathways in the low- and high-risk populations in the TCGA and CGGA cohorts. The results show that in the TCGA cohort, the high-risk group had a high level of immune cell infiltration, especially dendritic cells, B cells, macrophages, CD8+ T cells, helper T cells (Tfh, Th1, Th2), T tumour-infiltrating lymphocytes (TILs) and regulatory T (Treg) cells, compared to the lower-risk subgroup (Fig. 9C). The activity of the immune pathway was significantly upregulated in the high-risk group compared with the low-risk group (Fig. 9D). Similar conclusions were obtained in the CGGA cohort (Fig. 9E and F).

## Discussion

Tumours are generally considered to be the result of a series of genetic mutations that cause abnormal cell growth by activation of proto-oncogenes and inactivation of tumour suppressor genes. With the development and popularization of sequencing technologies in recent years, such as high-throughput sequencing technologies, researchers can more accurately understand the molecular characteristics of tumours at the genetic level [37]. The study of the mechanism of cell death can provide new ideas for the clinical treatment of tumours. Pyroptosis is a new type of programmed cell death discovered in recent years. It is essentially a cascade of inflammatory responses that plays an important role in immune defence and fighting infection [38, 39]. Previous studies have shown that the role of pyroptosis is mainly to eliminate bacterial and viral infections. The latest research has found that tumour cells will also undergo pyroptosis when there is no infection [40]. However, the mechanism of pyroptosis in tumours is still unclear.

In our study, we first evaluated the mRNA levels of 51 known pyroptosis-related genes in glioma and normal brain tissues and screened the genes that were differentially expressed. Subsequently, after further screening by the log-rank test, a total of 34 DEGs were retained for the next study. According to consensus clustering analysis, DEGs were divided into two clusters. The two clusters had significant differences in age and pathological grade. Survival analysis showed that the survival time of Cluster 1 was longer than that of Cluster 2. GSVA analysis showed that Cluster 1 was enriched in carcinogenic activation pathways, such as the WNT signalling pathway. Cluster 2 showed the enrichment of immune-related pathways, including ECM receptor interaction, complement and coagulation cascades, antigen processing and some immune-related diseases. To further evaluate the prognostic value of the DEGs, we constructed a 10-gene risk model through univariate Cox analysis and LASSO Cox regression analysis. Our results show that patients with high risk scores have a worse prognosis than patients with low risk scores. To further evaluate the accuracy of the prediction model, the CGGA database was used for verification. The results showed that the low-risk group in the validation set showed a better survival trend than the high-risk group. Univariate and multivariate Cox regression analyses were used to screen independent prognostic factors, and a nomogram was constructed for independent prognostic factors. The 1-year, 3-year, and 5-year OS calibration curves of the TCGA and CGGA datasets show similar performance to the ideal model, indicating that the nomogram has good prediction accuracy. KEGG enrichment analysis showed that DEGs between the low-risk and high-risk groups were related to multiple immune pathways. Finally, through the analysis of immune cell infiltration and activation pathways, we found that the level of immune cell infiltration in the high-risk group generally increased, and the activity of immune-related pathways increased compared with the low-risk group.

The occurrence of pyroptosis is divided into classical pathways and nonclassical pathways [11]. The classical pathway of pyroptosis depends on the activation of caspase-1. Intracellular receptors are stimulated by signals from bacteria, viruses, and cholesterol by recruiting apoptosis-associated speck-like protein to bind with the precursor of caspase-1, forming an inflammasome complex and then activating caspase-1 [41, 42]. Activated caspase-1 cleaves the gasdermin (GSDM) protein to form a lipophilic N-terminal domain, which interacts with the lipid layer of the cell membrane to form 10–20 nm pores to release inflammatory factors and expand the immune inflammatory response [43]. The nonclassical pathway of pyroptosis depends on caspase-11 and caspase-4/5. After cells are stimulated by bacterial lipopolysaccharide (LPS), caspase-4/5 and caspase-11 bind to bacterial LPS and are activated [44]. Activated caspase-4/5 and caspase-11 can also act on GSDMD and produce the same lysis effect as caspase-1, leading to cell membrane perforation [45].

Recent studies have shown that pyroptosis is closely related to the occurrence and development of tumours. Wang et al. showed that GSDMD, a key protein for pyroptosis, may inhibit the ERK, STAT3 and PI3K pathways, thereby inhibiting CyclinA2/CDK2, leading to cell cycle arrest and inhibiting the proliferation of gastric cancer [46]. Chen et al. reported that euxanthone can activate Caspase-1-dependent pyroptosis and significantly inhibit the proliferation, migration and invasion of liver cancer cells [47]. Our study produced a model with 10 pyroptosis-related genes (ELANE, TP63, GSDMC, IL18, IL1A, CASP3, CASP4, CASP9, CYCS and PLCG1) and found that it can predict the overall survival of patients with glioma. Scholars found that the ELANE gene may be related to the invasion and metastasis of clear cell renal cell carcinoma and is a potential prognostic and therapeutic marker of clear cell renal cell carcinoma [48]. TP63, a paralogue of TP73 and TP53, has been confirmed to be involved in the progression of a variety of tumours and has an impact on prognosis [49]. Wei J et al. found that the overexpression of GSDMC is an important factor in the poor prognosis of lung adenocarcinoma [50]. Caspase-1 can activate IL-1β and IL18 to cause pyroptosis [51]. Jiang et al. found that by regulating the expression of miRNA-214 in gliomas, the expression of caspase-1 was increased, leading to the secretion of IL-1β and IL-18 and promoting the occurrence and development of gliomas [52]. The latest research shows that caspase-3 can recognize and cleave the GSDMD-N-terminal domain, leading to the inhibition of pyroptosis [53]. Caspase-9 is currently a more in-depth study of the caspase promoter, which is closely related to proliferative diseases, degenerative diseases and cancer [54]. Kee-Beom et al. determined that PLCG1 is a key component of the amplified FGFR1 signal in small cell lung cancer and may become a potential target for disease treatment [55]. To date, although it has been found that there is an overlap between pyroptosis and apoptosis in the mechanism, pyroptosis has not been fully studied. With the development of tumour research, the coexistence of multiple cell death modes may be discovered. In our model, 7 genes (TP63, IL18, CASP3, CASP4, CASP9, CYCS and PLCG1) were also considered to be key regulators of the apoptosis pathway. Generally, apoptosis is characterized by pyknosis of the nucleus and the formation of apoptotic bodies, which will not cause inflammation, while pyroptosis is the opposite [56]. We analysed the differences in immune cell infiltration and immune-related pathways in different risk groups. Based on the analysis of ssGSEA, we speculate that pyroptosis can regulate the tumour immune microenvironment by causing inflammation. We preliminarily investigated the prognostic value of pyroptosis-related genes and provided theoretical support for future research. There are still some limitations in this study. Firstly, due to the heterogeneity of glioma tissue, more samples can be included in the future to ensure the stability and accuracy of signature prediction. Secondly, in the process of LASSO Cox regression analysis, some factors affecting the prognosis of glioma may be ignored. Thirdly, in our research, we found a pyroptosis-related gene signature to predict the prognosis of glioma through bioinformatics analysis. The data comes from public databases and lack more basic experimental verification. Finally, the complex interaction between gliomas and immune cells in pyroptosis remains to be further explored.

## Conclusion

Our research shows that there were differences in the expression of pyroptosis-related genes in gliomas. In the TCGA set, we constructed a risk model of 10 pyroptosis-related genes and found that the risk score is an independent predictor of glioma prognosis. The above results were verified in the CGGA validation set. Further research found that the difference between the low-risk group and the high-risk group was related to tumour immune infiltration. This study provides a new genetic marker for predicting the prognosis of patients with glioma and provides an important basis for further study of the relationship between pyroptosis-related genes and the immune microenvironment in glioma.

## Supplementary Information

**Additional file 1: Supplementary Table 1**. pyroptosis-related gene.

**Additional file 2: Supplementary Table 2**. GSVA analysis between the 2 cluster.

**Additional file 3: Supplementary Table 3**. KEGG analysis between high-risk and low-risk group.

## Data Availability

The data of this study were from The Cancer Genome Atlas (https://portal.gdc.cancer.gov/), the CGGA database (http://www.cgga.org.cn/index.jsp) and the xena database (http://xena.ucsc.edu/). Datasets generated for this study are included in the manuscript.

## Abbreviations

TCGA: The Cancer Genome Atlas
CGGA: Chinese Glioma Genome Atlas
DEGs: Differentially expressed genes
LASSO: Least absolute shrinkage and selection operator
GSEA: Gene Set Enrichment Analysis
ssGSEA: Single sample Gene Set Enrichment Analysis
GBM: Glioblastoma multiforme
LGG: Low grade glioma
OS: Overall survival
GSDM: Gasdermin
NCCD: Nomenclature Committee on Cell Death
HIV: Human immunodeficiency virus
GDC: Genomic Data Commons
GTEx: Genotype-Tissue Expression
FDR: False discovery rate
GO: Gene Ontology
KEGG: Kyoto Encyclopedia of Genes and Genomes
ROC: Receiver operating characteristic curve
AUC: Area under the curve
PCA: Principal component analysis
