# Supplementary material for: A novel pyroptosis-related gene signature predicts the prognosis of glioma through immune infiltration
Source: BMC Cancer. 2021 Dec 7;21:1311. doi: 10.1186/s12885-021-09046-2 (PMC8653573; doi:10.1186/s12885-021-09046-2)
Supplement: Supplementary file 2 — Additional file 2: Supplementary Table 2. GSVA analysis between the 2 cluster. [file 12885_2021_9046_MOESM2_ESM.docx]

| ID | adj.P.Val |
| --- | --- |
| KEGG_COMPLEMENT_AND_COAGULATION_CASCADES | 5.99E-96 |
| KEGG_SYSTEMIC_LUPUS_ERYTHEMATOSUS | 8.40E-96 |
| KEGG_GLYCOSAMINOGLYCAN_DEGRADATION | 1.90E-94 |
| KEGG_INTESTINAL_IMMUNE_NETWORK_FOR_IGA_PRODUCTION | 2.98E-93 |
| KEGG_ALLOGRAFT_REJECTION | 5.97E-92 |
| KEGG_GRAFT_VERSUS_HOST_DISEASE | 9.00E-89 |
| KEGG_PRIMARY_IMMUNODEFICIENCY | 8.83E-86 |
| KEGG_AMINO_SUGAR_AND_NUCLEOTIDE_SUGAR_METABOLISM | 1.04E-85 |
| KEGG_AUTOIMMUNE_THYROID_DISEASE | 2.40E-84 |
| KEGG_GLUTATHIONE_METABOLISM | 4.99E-83 |
| KEGG_LEISHMANIA_INFECTION | 4.02E-82 |
| KEGG_HEMATOPOIETIC_CELL_LINEAGE | 8.74E-79 |
| KEGG_CYTOKINE_CYTOKINE_RECEPTOR_INTERACTION | 4.90E-76 |
| KEGG_ASTHMA | 2.09E-74 |
| KEGG_TYPE_I_DIABETES_MELLITUS | 4.27E-74 |
| KEGG_ANTIGEN_PROCESSING_AND_PRESENTATION | 7.42E-74 |
| KEGG_ECM_RECEPTOR_INTERACTION | 6.88E-73 |
| KEGG_TASTE_TRANSDUCTION | 1.63E-69 |
| KEGG_LONG_TERM_DEPRESSION | 2.10E-68 |
| KEGG_WNT_SIGNALING_PATHWAY | 6.83E-67 |

Supplementary Table 2. GSVA analysis between the 2 cluster.
