# Supplementary material for: A novel pyroptosis-related gene signature predicts the prognosis of glioma through immune infiltration
Source: BMC Cancer. 2021 Dec 7;21:1311. doi: 10.1186/s12885-021-09046-2 (PMC8653573; doi:10.1186/s12885-021-09046-2)
Supplement: Supplementary file 3 — Additional file 3: Supplementary Table 3. KEGG analysis between high-risk and low-risk group. [file 12885_2021_9046_MOESM3_ESM.docx]

| ID | Description | p.adjust |
| --- | --- | --- |
| hsa04933 | AGE-RAGE signaling pathway in diabetic complications | 0.000145092 |
| hsa04926 | Relaxin signaling pathway | 0.000321367 |
| hsa04512 | ECM-receptor interaction | 0.000412761 |
| hsa04145 | Phagosome | 0.000412761 |
| hsa05150 | Staphylococcus aureus infection | 0.000483379 |
| hsa04974 | Protein digestion and absorption | 0.000566549 |
| hsa04510 | Focal adhesion | 0.001139516 |
| hsa05322 | Systemic lupus erythematosus | 0.001603949 |
| hsa05140 | Leishmaniasis | 0.001937382 |
| hsa05310 | Asthma | 0.001950807 |
| hsa04658 | Th1 and Th2 cell differentiation | 0.00277701 |
| hsa05330 | Allograft rejection | 0.00277701 |
| hsa05323 | Rheumatoid arthritis | 0.00277701 |
| hsa05332 | Graft-versus-host disease | 0.00324843 |
| hsa05146 | Amoebiasis | 0.00324843 |
| hsa04940 | Type I diabetes mellitus | 0.00324843 |
| hsa04672 | Intestinal immune network for IgA production | 0.004496368 |
| hsa05320 | Autoimmune thyroid disease | 0.005345766 |
| hsa05165 | Human papillomavirus infection | 0.006165547 |
| hsa05416 | Viral myocarditis | 0.006906182 |
| hsa04151 | PI3K-Akt signaling pathway | 0.007888037 |
| hsa05321 | Inflammatory bowel disease | 0.007914519 |
| hsa04612 | Antigen processing and presentation | 0.012764201 |
| hsa05164 | Influenza A | 0.014802197 |
| hsa04610 | Complement and coagulation cascades | 0.01498227 |
| hsa05152 | Tuberculosis | 0.016080127 |
| hsa04657 | IL-17 signaling pathway | 0.018409216 |
| hsa04640 | Hematopoietic cell lineage | 0.020514784 |

Supplementary Table 3. KEGG analysis between high-risk and low-risk group.
